# Keeping the Teeth Clean

**Published:** 1883-05

**Authors:** C. E. Francis


ARTICLE VII.

### KEEPING THE TEETH CLEAN.

C. E. FRANCIS, D, D. S., M. D. S., N. Y.

It is a deplorable fact that the mass of mankind are culpably negligent in caring for their teeth.

Useful as are these organs as aids in the promotion of health, comfort, and longevity, they are often sadly abused, and as a consequence, not infrequently do they prove rebellious and become a source of dire annoyance.

Many people defer visiting a dentist until driven by relentless pain to seek relief, after having vainly exhausted the various domestic remedies suggested by sympathizing friends. By that time, in all probability, the offending member and perhaps several others are found to be in an exceedingly dilapidated condition; possibly ruined. In such cases very likely all the remaining teeth have become badly stained or coated with incrustations of salivary calculus; with gums purple and humid, and ready to bleed at the slightest touch.

Some mouths, so far as the invasion of a tooth brush is concerned, are unexplored caverns of a miniature type; and others which receive but an occasional visit from this intrusive explorer, are not in a much better condition for the little care bestowed upon them.

But there are many, very many well meaning individuals who habitually brush their teeth, and some even declare that they perform this duty twice, thrice or four times daily, yet cannot keep their teeth from becoming stained or covered with "tartar."

Who has not witnessed cases where the teeth, after having received a most thorough cleansing by a dentist, have within a few months after, been again covered with accumulations as repulsive to the eye as if they had never been cleansed? And yet, when expressions of surprise follow such discoveries, assurance is given that the tooth-brush is regularly used!

It is certainly disheartening to a dentist who, after having taxed his best efforts to save from total destruction a set of teeth nearly wrecked by abuse or neglect, to subsequently find them again stamped with stains, and their interstices loaded with extraneous matter.

On the principle that "like causes produce like results." teeth ever so skillfully treated by the dentist, if in this manner are constantly menaced by invasions from such mischievous elements of decalcification, what wonder is it if fillings occasionally become undermined with decay and prove failures?

"Why cannot I keep my teeth free from 'tartar'?" is a question frequently asked by discouraged patients. "It is not from lack of brushing," they say. To express a doubt as to *thoroughness* on their part is a delicate thing to do, yet proofs are sometimes painfully apparent to warrant such a doubt. Undoubtly many individuals imagine they are particular in this respect when they are not.

The fact is, very few persons know how to properly manipulate a brush; nor do they know what sort of a brush to select. Scarcely one in ten of the brushes manufactured are fit for use, and this statement is no exaggeration. Many are too large and unwieldly to be successfully managed, and would be more suitable for "nail-brushing." The majority of them was also too compact; some too rigid and not sufficiently pliable to be useful, while others are too soft and little better than rags. The brush for service should never be broader than the medium sizes usually sold, nor over two-thirds their length. The bristles should be elastic and their ends trimmed in serratisns, or "notched"—this form being best adapted to the shape of the teeth.

In use the brush should be pressed firmly against the teeth commencing with the back ones at their cervical borders, and with a semirotary motion slowly brought forward and towards their grinding edges in such a manner as to force from between them accumulations that have found lodgement there; also allowing the bristles to come in contact with all enamel surfaces possible to reach.

Rapid horizontal dashes should be avoided. A brush furiously across the teeth touches only points of enamel that least require rubbing, leaving the accumulations that load their interstices undisturbed or unmolested.

It is not the frequency of brushing that best preserves the teeth, but the degree of thoughtfulness with which it is done. The time for performing this duty most effectively is just before retiring for the night. During the twelve hours interval from the evening meal to the morning repast, particles of food retained about the teeth and subjected to the warm humid condition of the oral cavity, cannot fail to decomposed or fermented, thus breeding an insiduous foe that, night after night, besieges the canal walls which, unless of extraordinary compactness, will sooner or later give way to its destructive forces.

There is no objection to cleansing the teeth when making the morning toilet, yet if thoroughly cared for the night before, they require comparatively little of such attention in the early part of the day. To brush them more frequently than this is a needless task.

"Prevention" being considered better that "cure," it would seem an important part of the dentist's duty to give such instruction to his patients as will enable them to keep their teeth in a condition of cleanliness.

How many are sufficiently particular in this respect? —*Independent Practitioner.*

---

ARTICLE VIII.

